# Bcl‐2 hijacks the arsenic trioxide resistance in SH‐SY5Y cells

**DOI:** 10.1111/jcmm.17128

**Published:** 2021-12-14

**Authors:** Jinling Wang, Xiaohui Peng, Daowei Yang, Mengyu Guo, Xiao Xu, Fengyue Yin, Yu Wang, Jiaqing Huang, Linghui Zhan, Zhongquan Qi

**Affiliations:** ^1^ Department of Emergency Zhongshan Hospital of Xiamen University Xiamen China; ^2^ Department of General Surgery Xiamen Fifth Hospital Xiamen China; ^3^ Department of Clinical Sciences Malmö, Lund University Malmö Sweden; ^4^ Medical College of Guangxi University Nanning China; ^5^ Department of Intensive Care Medicine Zhongshan Hospital of Xiamen University Xiamen China

**Keywords:** arsenic trioxide (ATO), Bcl‐2, mitochondrial morphology, resistance

## Abstract

Aresenic trioxide (ATO) is proven to be active against leukaemia cells by inducing apoptosis and differentiation. Even though ATO could effectively induce remissions of leukaemia cells, the drug resistance was observed occasionally. To further dissect the mechanism of ATO resistance, we selected the ATO‐resistant SH‐SY5Y cells and found that Bcl‐2 controlled the sensitivity of ATO in SH‐SY5Y cells. We report that necroptosis, autophagy, NF‐ƘB and MAPK signalling pathway are not involved in ATO‐induced apoptosis. Moreover, the ATO‐resistant cells showed distinct mitochondrial morphology compared with that of ATO‐sensitive cells. Intriguingly, nude mice‐bearing ATO‐sensitive cells derived xenograft tumours are more sensitive to ATO treatment compared with that of ATO‐resistant cells. These data demonstrate that cancer cells can acquire the ATO‐resistance ability by increasing the Bcl‐2 expression.

## 1 INTRODUCTION

ATO is a marvellously effective drug used for the treatment of acute promyelocytic leukaemia (APL).^1^ In APL, one of its anti‐cancer mechanisms is via degradation of the PML‐RAR fusion protein, inhibition of cancer stem cells and induction of apoptosis.^2^ It was suggested that the combinational administration of ATO with other potential drugs has promising usage in increasing drug efficacy and decreasing the drug resistance.^3^

Apoptosis is a form of programmed cell death, which involves in normal organ development and immunity.^4^ ATO‐induced apoptosis in lung cancer cells is related to increased Fas expression.^5^ Moreover, it has been revealed that ATO can increase the effect of tumour necrosis factor‐related apoptosis‐inducing ligand (TRAIL) and inhibit the expression of NF‐kB to induce apoptosis.^6^, ^7^

ATO can directly bind the promyelocytic leukaemia protein (PML) B2 domain to suppress the progression of APL.^8^ Mutation of PML‐C212/213 has been reported to result in the resistance of ATO in an ex vivo model.^8^ Importantly, directly detected amino acid substitutions of A216V and L218P in patients with APL have been validated to be resistant to ATO.^9^

Although studies involving the efficacy of ATO have been validated in many cancer cells, the ATO resistance is still inadequate and further studies are needed to investigate. It is likely that anti‐apoptosis pathways are altered in ATO resistance. Herein, we generated and evaluated the ATO‐resistant SY‐SY5Y clones and demonstrated that ATO‐resistant cells acquired the ATO‐resistance ability by increasing the Bcl‐2 expression.

## 2 MATERIALS AND METHODS

### 2.1 Cell culture and reagents

SH‐SY5Y cells were obtained from ATCC. All cells were cultured in DMEM supplemented with 10% FBS and non‐essential amino acids at 37°C in a humidified incubator containing 5% CO_2_. The BCL‐2, BCL‐xL, AIF and BAX antibodies were obtained from Abcam. Caspase‐3 antibody (9662S) was obtained from Cell Signaling Technology. GAPDH and β‐actin antibodies were obtained from Proteintech. The LDH cytotoxicity assay kit (J2380) was obtained from Promega. ATP Cell Titer‐Glo assay kit was obtained from Promega. The Mitoview 633 and DAPI were obtained from Biotium.

### 2.2 Plasmids construction

Bcl‐2 sequence was amplified from human cDNA and cloned into Pbobi lentivirus vector with N‐terminally tagged as previously described.^10^

### 2.3 Confocal microscopy

SH‐SY5Y cells were grown on coverslips (Nest) in 12‐well plates. Cells were then washed with PBS followed by incubated with Mitoview 633 for 0.5 h. Then, the cells were stained with Hoechst to visualize nucleus. Imaging was performed by using Zeiss LSM 780. All images were processed and quantified by ImageJ software.

### 2.4 Cell survival and cytotoxicity assay

LDH assay was performed using CytoTox 96 Non‐Radioactive Cytotoxicity Assay kit from Promega Corporation. Cell survival assay was performed using Cell Titer‐Glo Luminescent Cell Viability Assay kit as previously described.^10^ All these experiments were performed according to the manufacturer's instructions.

### 2.5 RNA sequencing

P cells and D cells were cultured for 24 h; then, total RNA was extracted with an RNA extraction kit (Qiagen) and used for library generation. The samples were submitted to Novogene for RNA sequencing. The libraries were sequenced on the Illumina NovaSeq 6000 platform. All the data were analysed with Genepix software (Molecular Devices USA).

### 2.6 Mouse xenograft models

Four‐ to six‐week‐old female BALB/c nude mice were obtained from Xiamen University Animal Center. To establish the tumour xenograft mouse models, 5 × 10^6^ P cells or D cells in 200 μl saline were implanted subcutaneously into the right flank of the nude mice. After implantation, tumour volumes were measured with a slide calliper every 2 days and calculated using the following formula: 0.5 × (length) × (width)^2^. Then, the tumour‐bearing mice were divided randomly into two groups (6 mice in each group) and treated with 10 mg/kg ATO every 2 days by i.p. injection. The mice were sacrificed after six times injection, and tumour tissues were excised, weighed and photographed. Subsequently, xenograft tumour and the major organs were fixed in neutral‐buffered formalin for Ki67 staining according to the manufacture's instruction. All of the animals were treated according to protocols approved by the Institutional Animal Care and Use Committee of the Xiamen University.

### 2.7 Immunoblotting

Western blotting was performed as previously described.^11^ Proteins were separated by SDS‐PAGE and transferred to PVDF membrane. The membrane was blocked with 5% BSA and incubated at 4°C overnight with indicated first antibodies, followed by incubation with corresponding HRP‐conjugated secondary antibodies. The protein bands were detected with an ECL.

### 2.8 Statistical analysis

All results are analysed using the GraphPad 6.0 software and expressed as means ± standard error of the mean (SEM). All experiments were performed using at least three independent biological replicates. For two group compassion, an unpaired two‐tailed Student's *t* test was used.

## 3 RESULTS

### 3.1 Arsenic trioxide (ATO) can induce apoptosis in SH‐SY5Y cells and necroptosis, autophagy, NF‐ƘB and MAPKs signalling pathways are not involved in ATO‐induced apoptosis

**FIGURE 1:**
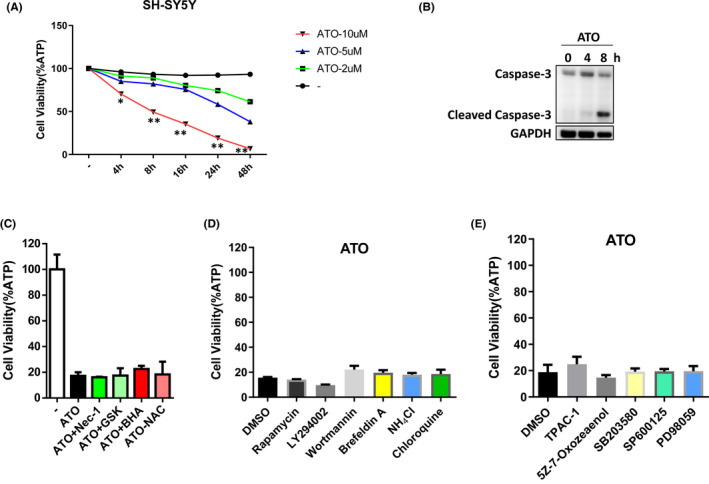
Arsenic trioxide (ATO) can induce apoptosis in SH‐SY5Y cells, and necroptosis, autophagy, NF‐ƘB and MAPKs signalling pathways are not involved in ATO‐induced apoptosis. (A) SH‐SY5Y cells were treated with indicated concentration of ATO for different hours. Cell survival was determined by measuring ATP levels. Results are representative of three independent experiments. (B) SH‐SY5Y cells were treated for indicated hours with 10 µM ATO. The cells were lysed and immunoblotted with caspase‐3 and GAPDH antibodies. (C–E) SH‐SY5Y cells were treated with indicated reagents. Cell survival was determined by measuring ATP levels. Results are representative of three independent experiments. *p < 0.05, **p < 0.01, compared between the cells treated with 10 µM ATO and Ctrl group

ATO has demonstrated a dramatic clinical effect on acute promyelocytic leukaemia (APL) patients. ATO exhibits its antitumour effect on various tumour cells by causing apoptosis.^12^ To explore the efficacy of ATO in SH‐SY5Y neuroblastoma cells, we treated the cells with different concentrations of ATO for different hours. The results revealed that treatment with 10 µM ATO‐induced significant cell death (Figure 1A) and ATO increased the levels of cleaved caspase‐3 (Figure 1B). Thus, our results showed that ATO can induce apoptosis in SH‐SY5Y cells.

Three classical forms of cell death are composed of apoptosis, autophagy and necroptosis^13^; thus, forms of cell death reveal distinct morphological characteristics by inducing different signalling pathways. To explore whether other forms of cell death are involved in ATO‐induced cell death, we treated the SH‐SY5Y cells with the inhibitors of necroptosis and autophagy. However, four known necroptosis inhibitors, Nec‐1, GSK’862, BHA and NAC, could not influence ATO‐induced cell death (Figure 1C). Meanwhile, several autophagy inhibitors, such as rapamycin, wortmannin and chloroquine, could not affect ATO‐induced cell death (Figure 1D). Previous reports suggested that the inhibition of MAPK and NF‐kB pathways triggered apoptosis,^14^ so we used the inhibitors of MAPK and NF‐kB signalling pathways to test the effect of such survival pathways on ATO‐induced cell death. However, MAPK or NF‐kB pathway inhibitors (eg TPCA‐1 and PD98059) did not display any function in ATO‐induced cell death (Figure 1E). Collectively, these results show that necroptosis, autophagy, NF‐ƘB and MAPKs signalling pathways are not involved in ATO‐induced apoptosis.

### 3.2 P cells are sensitive to ATO‐induced cell death and have distinct mitochondrial features after ATO treatment compared with D cells

**FIGURE 2:**
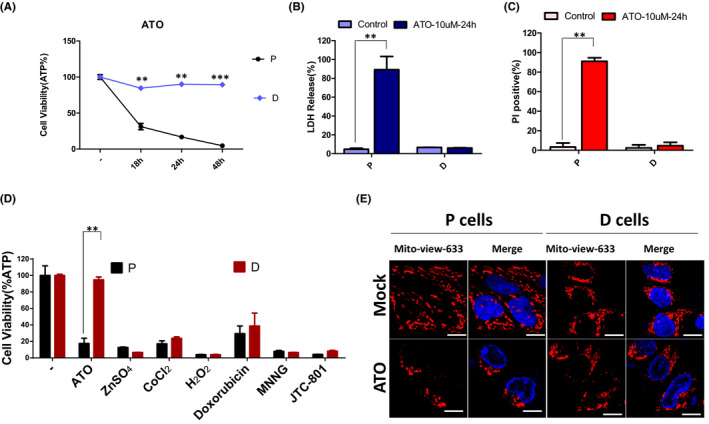
P cells are sensitive to ATO‐induced cell death and have distinct mitochondrial features after ATO treatment compared with D cells. (A) P cells and D cells were treated with 10 µM ATO for indicated hours. Cell survival was determined by measuring ATP levels. (B) P cells and D cells were treated with 10 µM ATO for 24 h, and the LDH release was measured by the LDH activity assay kit. (C) P cells and D cells were treated with 10 µM ATO for 24 h, and the PI positive was determined by PI staining. (D) P cells and D cells were treated with indicated reagents. Cell survival was determined by measuring ATP levels. Results are representative of three independent experiments. **p < 0.01 and ***p < 0.001 compared between the P cells and D cells or compared with the control group. (E) P cells and D cells were treated with 10 µM ATO for 18 h, and the mitochondrial morphology was determined by Mitoview‐633 staining and the nucleus was stained by Hoechst. Scale bars, 10 µm

To dissect the mechanisms governing the ATO resistance in cancer cells, we seeded the single SH‐SY5Y cell into the 96‐well plate to get the different genetic background single clone of SH‐SY5Y cells. Afterwards, we tested the ATO sensitivity of above single clones and got the ATO‐sensitive cells (hereafter named P cells) and ATO‐resistant cells (hereafter named D cells) (Figure 2A). Moreover, we found that P cells were more sensitive than D cells in the lactate dehydrogenase (LDH) release (Figure 2B) and propidium iodide (PI) penetration (Figure 2C). To ascertain the different responses of P cells and D cells is ATO specific, ATO, ZnSO_4_, CoCl_2_, H_2_O_2_, Doxorubicin, MNNG and JTC‐801 were used in parallel to stimulate P cells and D cells. The results were shown as in Figure 2D, and the different responses of P cells and D cells were only revealed in the process of ATO treatment. Taken together, our results suggest that P cells and D cells are the ideal models of ATO resistance.

Mitochondria has dynamic networks that change shape and subcellular localization.^15^, ^16^ It was reported that dramatic alterations in mitochondrial morphology in the process of apoptosis, and mitochondrial fragmentation was often described in connection with many modes of apoptosis.^15^ Importantly, proteins that controlled mitochondrial morphology seem to also regulate the progression of apoptosis. To understand the mechanisms governing the ATO resistance in the D cells, we compared the difference in mitochondrial morphology between P cells and D cells. The results showed that degradation of the tubular mitochondria and formation of punctiform occurred in the P cells treated with ATO for 24 h, whereas the fragmented mitochondria could not be observed in D cells (Figure 2E). These data demonstrate that consistent mitochondrial morphology possesses a correlation with the ATO resistance in D cells.

### 3.3 Bcl‐2 controls the ATO resistance in D cells

**FIGURE 3:**
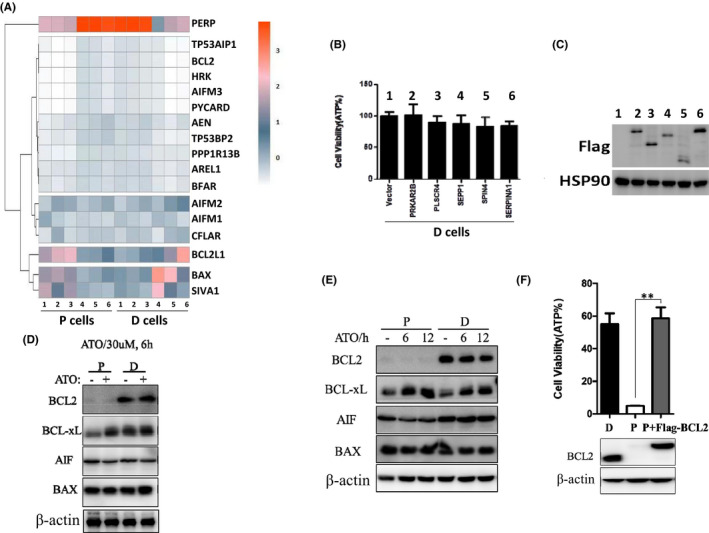
Bcl‐2 controls the ATO resistance in D cells. (A) Microarry analysis of different cell lines. Heatmap of genes upregulated or downregulated in different cell lines was shown. (B) D cells were transfected with indicated genes decreased, then these cells were treated with 10 µM ATO for 24 h, and the cell variability was measured by ATP level. (C) Western blotting was used to detect indicated protein in the cells as shown in (B). (D) P cells and D cells were treated with or without 30 µM ATO for 6 h, and the cells were lysed and immunoblotted with indicated antibodies. (E) P cells and D cells were treated with or without 10 µM ATO for 6 h and 12 h; then, the cells were lysed and immunoblotted with indicated antibodies. (F) Upper: P cells with or without overexpression of Bcl‐2 and D cells were treated with 10 µM ATO for 24 h. Cell survival was determined by measuring ATP levels. Bottom: The above cells were lysed and immunoblotted with indicated antibodies. **p < 0.01, compared between the P cells and P cells overexpressed Bcl‐2

Using RNA‐Seq, we compared mRNAs that are significantly different between P cells and D cells (Figure 3A). Among these genes, we found that *PRKAR2B*, *PLSCR4*, *SEPP1*, *SPIN4* and *SERPINA1* were dramatically decreased in D cells. Then, we tried to overexpress these decreased genes in D cells and stimulated the cells with ATO for 24 h. However, we could not find any difference in D cells overexpressed with above genes compared with the control cells after ATO treatment (Figure 3B). We also checked the protein expression level in D cells and found all the genes expressed correctly (Figure 3C). Taken together, these data suggest that the decreased genes do not confer the resistance ability of D cells.

Considering mitochondrial outer membrane permeabilization is regulated by Bcl‐2 family,^17^ and the RNA‐seq data also suggested that Bcl‐2 was decreased in P cells. Then, the expression of Bcl‐2 and Bcl‐xL was examined in the P cells and D cells treated with ATO for different hours. We found that levels of Bcl‐2 increased in the D cells and Bcl‐2 was hardly detected in P cells, whereas the levels of Bcl‐xL, AIF and BAX were consistent (Figure 3D,E). To validate the increased Bcl‐2 controlled the ATO resistance of D cell, we overexpressed Bcl‐2 in the P cells and got the same levels of Bcl‐2 between P cells and D cells. The cell viability data suggested that P cells with overexpressed Bcl‐2 became resistant to ATO‐induced apoptosis (Figure 3F). Taken together, these results suggest that Bcl‐2 impedes ATO‐induced cell death and the levels of Bcl‐2 controls the ATO resistance in D cells.

### 3.4 ATO plays a more dramatic function in inhibiting the tumorigenesis of P cells

**FIGURE 4:**
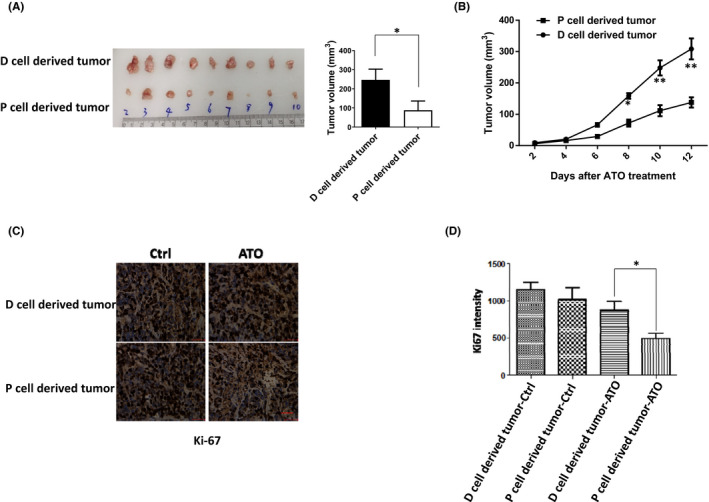
ATO plays a dramatic function in inhibiting the tumorigenesis of P cells. (A) Nude mice‐bearing P cells and D cells xenograft tumours for 12 days, and ATO was administered by i.p. injection once at 10 mg/kg every two days, six times in total. Then, the mice were sacrificed and tumours were removed, photographed and measured. (B) After implantation, tumour volumes were measured with a slide calliper every two days. (C) Tumours as shown in (A) were used to do the Ki67 immunostaining, and the intensity of Ki67 was measured and shown in (D), scale bar, 100 µm. *p < 0.05 and **p < 0.01, compared between the P cells and D cells

To determine whether P cells and D cells exhibited different responses to ATO treatment *in vivo*, we injected P cells and D cells subcutaneous injection into nude mice. After implantation, ATO was administered to tumour‐bearing mice by intraperitoneal injection once every other day. Twelve days later, the mice were sacrificed and tumour tissues were excised, photographed and weighed. As anticipated, ATO inhibited the growth of P cells derived xenograft tumour more significantly than that of D cells (Figure 4A,B). To confirm this result, we also did the Ki67 staining, a cellular marker for proliferation, and the Ki67 staining result was consistent with the volume of tumour (Figure 4C,D). Thus, these data suggest that the D cells were more resistant to ATO treatment *in vivo* and ATO played a more dramatic function in inhibiting the tumorigenesis of P cells.

## 4 DISCUSSION

Bcl‐2 has a pivotal role in regulating mitochondrial apoptosis. Downregulation of Bcl‐2 can result in mitochondrial outer membrane permeabilization (MOMP) and the release of cytochrome c. Recent studies have demonstrated that ATO induces apoptosis by reducing Bcl‐2 protein in lung cancer. Therefore, the level of Bcl‐2 has significant importance for ATO‐induced apoptosis in cancer cells.

Apoptosis is an efficient way to exert anti‐cancer function after ATO treatment; however, many cancer cells can acquire resistance ability after long‐term ATO stimulation. Some rare genetic mutations maybe confer the ATO resistance, but the exact mechanisms need to be explored. In addition, the severe side effect was reported in patients with lung cancer after high dose of ATO treatment.^18^ Recently, pyroptosis was reported in U251 cells after ATO treatment and this kind of cell death was more potent to induce immunity response to diminish tumour.^19^, ^20^ Therefore, it is possible to combine different drugs with ATO to enhance pyroptosis in cancer cells; thus, the low dose of ATO will not trigger the side effect in patients.

Many factors can determine the ATO sensitivity, and the dominant underlying mechanism of ATO resistance is still elusive. Most clinical researches focused on the *PML* B2 domain mutations in patients who acquired ATO resistance.^21^ However, more and more different genetic mutations were predicted to have the relation with ATO resistance and disease recurrence.^22^ In comparison with the genetic variants, we characterized at the Bcl‐2 protein level to demonstrate the cancer cells can acquire resistance ability by increasing the Bcl‐2 expression. Novel agents target Bcl‐2 and combinatorial treatment based on these findings are needed to solve the issue of ATO resistance in patients with relapsed APL.

## CONFLICT OF INTEREST

The authors have no financial or proprietary interests in any material discussed in this article.

## AUTHOR CONTRIBUTIONS

**Jinling Wang:** Conceptualization (equal); data curation (equal); investigation (equal); methodology (equal); project administration (equal); resources (equal); supervision (equal). **Xiaohui Peng:** Investigation (equal); methodology (equal). **Daowei Yang:** Conceptualization (equal); investigation (equal); methodology (equal); project administration (equal); visualization (equal); writing – original draft (equal); writing – review & editing (equal). **Mengyu Guo:** Data curation (equal); investigation (equal); methodology (equal). **Xiao Xu:** Data curation (equal); investigation (equal); methodology (equal). **Fengyue Yin:** Data curation (equal); investigation (equal); methodology (equal). **Yu Wang:** Data curation (equal); investigation (equal); methodology (equal). **Jiaqing Huang:** Data curation (equal); investigation (equal); methodology (equal). **Linghui Zhan:** Investigation (equal); project administration (equal); software (equal). **Zhongquan Qi:** Conceptualization (lead); data curation (lead); funding acquisition (equal); investigation (lead); methodology (lead); project administration (equal); resources (lead); validation (lead); writing – original draft (lead).

## Data Availability

The datasets generated during the current study are available from the corresponding author on reasonable request.

## References

[1] Chen GQ , Shi XG , Tang W , et al. Use of arsenic trioxide (As2O3) in the treatment of acute promyelocytic leukemia (APL). 1. As2O3 exerts dose‐dependent dual effects on APL cells. Blood. 1997;89:3345‐3353. 9129041

[2] Wang ZY , Chen Z . Acute promyelocytic leukemia: from highly fatal to highly curable. Blood. 2008;111:2505‐2515. 18299451 10.1182/blood-2007-07-102798

[3] Tian W , Wang Z , Tang NN , et al. Ascorbic acid sensitizes colorectal carcinoma to the cytotoxicity of arsenic trioxide via promoting reactive oxygen species‐dependent apoptosis and pyroptosis. Front Pharmacol. 2020;11:123. 32153415 10.3389/fphar.2020.00123 PMC7047232

[4] Elmore S . Apoptosis: a review of programmed cell death. Toxicol Pathol. 2007;35:495‐516. 17562483 10.1080/01926230701320337 PMC2117903

[5] Yang GF , Li XH , Zhao Z , Wang WB . Arsenic trioxide up‐regulates Fas expression in human osteosarcoma cells. Chin Med J‐Peking. 2010;123:1768‐1773. 20819644

[6] Kerbauy DM , Lesnikov V , Abbasi N , Seal S , Scott B , Deeg HJ . NF‐kappaB and FLIP in arsenic trioxide (ATO)‐induced apoptosis in myelodysplastic syndromes (MDSs). Blood. 2005;106:3917‐3925. 16105982 10.1182/blood-2005-04-1424 PMC1895102

[7] Wang JY , Zhao XQ , Wang CM , Mo BW , Jiang M , Chen F . Arsenic trioxide enhances TRAIL inducing human lung cancer cell line A549 cells apoptosis by down‐regulate the expression of NF‐kappaB. Sichuan Da Xue Xue Bao Yi Xue Ban. 2012;43:834‐838. 23387208

[8] Noguera NI , Catalano G , Banella C , et al. Acute promyelocytic leukemia: update on the mechanisms of leukemogenesis, resistance and on innovative treatment strategies. Cancers. 2019;11(10):1591. 10.3390/cancers11101591 PMC6826966 31635329

[9] Zhu HH , Qin YZ , Huang XJ . Resistance to arsenic therapy in acute promyelocytic leukemia. New Engl J Med. 2014;370:1864‐1866. 24806185 10.1056/NEJMc1316382

[10] Yang D , Liang Y , Zhao S , et al. ZBP1 mediates interferon‐induced necroptosis. Cell Mol Immunol. 2020;17:356‐368. 31076724 10.1038/s41423-019-0237-x PMC7109092

[11] Wang J , Yang D , Shen X , et al. BPTES inhibits anthrax lethal toxin‐induced inflammatory response. Int Immunopharmacol. 2020;85:106664. 32521490 10.1016/j.intimp.2020.106664

[12] Fang Y , Zhang Z . Arsenic trioxide as a novel anti‐glioma drug: a review. Cell Mol Biol Lett. 2020;25:44. 32983240 10.1186/s11658-020-00236-7 PMC7517624

[13] Kroemer G , Galluzzi L , Vandenabeele P , et al. Classification of cell death: recommendations of the Nomenclature Committee on Cell Death 2009. Cell Death Differ. 2009;16:3‐11. 18846107 10.1038/cdd.2008.150 PMC2744427

[14] Papademetrio DL , Lompardia SL , Simunovich T , et al. Inhibition of survival pathways MAPK and NF‐kB triggers apoptosis in pancreatic ductal adenocarcinoma cells via suppression of autophagy. Target Oncol. 2016;11:183‐195. 26373299 10.1007/s11523-015-0388-3

[15] Karbowski M , Youle RJ . Dynamics of mitochondrial morphology in healthy cells and during apoptosis. Cell Death Differ. 2003;10:870‐880. 12867994 10.1038/sj.cdd.4401260

[16] Lackner LL . Shaping the dynamic mitochondrial network. Bmc Biol. 2014;12:35. 24884775 10.1186/1741-7007-12-35 PMC4035697

[17] Kale J , Osterlund EJ , Andrews DW . BCL‐2 family proteins: changing partners in the dance towards death. Cell Death Differ. 2018;25:65‐80. 29149100 10.1038/cdd.2017.186 PMC5729540

[18] Huang W , Zeng YC . A candidate for lung cancer treatment: arsenic trioxide. Clin Transl Oncol. 2019;21:1115‐1126. 30756240 10.1007/s12094-019-02054-6

[19] Wang QY , Wang YP , Ding JJ , et al. A bioorthogonal system reveals antitumour immune function of pyroptosis. Nature. 2020;579(7799):421‐426. 32188939 10.1038/s41586-020-2079-1

[20] Wang JL , Zhan LH , Cai Z , et al. Arsenic trioxide induces gasdermin E mediated pyroptosis in astroglioma cells. Transl Cancer Res. 2020;9:1926‐1930. 10.21037/tcr.2020.02.17 PMC8797398 35117539

[21] Tomita A , Kiyoi H , Naoe T . Mechanisms of action and resistance to all‐trans retinoic acid (ATRA) and arsenic trioxide (As2O3) in acute promyelocytic leukemia. Int J Hematol. 2013;97:717‐725. 23670176 10.1007/s12185-013-1354-4

[22] Weinhauser I , Pereira‐Martins DA , Ortiz C , et al. Reduced SLIT2 is associated with increased cell proliferation and arsenic trioxide resistance in acute promyelocytic leukemia. Cancers. 2020;12(11):3134. 10.3390/cancers12113134 PMC7693375 33120864

